# Skeletal Muscle-Derived Exosomal miR-146a-5p Inhibits Adipogenesis by Mediating Muscle-Fat Axis and Targeting GDF5-PPARγ Signaling

**DOI:** 10.3390/ijms24054561

**Published:** 2023-02-25

**Authors:** Mengran Qin, Lipeng Xing, Jiahan Wu, Shulei Wen, Junyi Luo, Ting Chen, Yaotian Fan, Jiahao Zhu, Lekai Yang, Jie Liu, Jiali Xiong, Xingping Chen, Canjun Zhu, Songbo Wang, Lina Wang, Gang Shu, Qingyan Jiang, Yongliang Zhang, Jiajie Sun, Qianyun Xi

**Affiliations:** 1 Guangdong Provincial Key Laboratory of Animal Nutrition Control, State Key Laboratory of Livestock and Poultry Breeding, National Engineering Research Center for Breeding Swine Industry, College of Animal Science, South China Agricultural University, No. 483 Wushan Road, Guangzhou 510642, China; 2 Jiangxi Province Key Laboratory of Animal Nutrition, College of Animal Science and Technology, Jiangxi Agricultural University, Nanchang 330045, China

**Keywords:** skeletal muscle, exosomes, miR-146a-5p, adipogenesis, GDF5, crosstalk, PPARγ

## Abstract

Skeletal muscle-fat interaction is essential for maintaining organismal energy homeostasis and managing obesity by secreting cytokines and exosomes, but the role of the latter as a new mediator in inter-tissue communication remains unclear. Recently, we discovered that miR-146a-5p was mainly enriched in skeletal muscle-derived exosomes (SKM-Exos), 50-fold higher than in fat exosomes. Here, we investigated the role of skeletal muscle-derived exosomes regulating lipid metabolism in adipose tissue by delivering miR-146a-5p. The results showed that skeletal muscle cell-derived exosomes significantly inhibited the differentiation of preadipocytes and their adipogenesis. When the skeletal muscle-derived exosomes co-treated adipocytes with miR-146a-5p inhibitor, this inhibition was reversed. Additionally, skeletal muscle-specific knockout miR-146a-5p (mKO) mice significantly increased body weight gain and decreased oxidative metabolism. On the other hand, the internalization of this miRNA into the mKO mice by injecting skeletal muscle-derived exosomes from the Flox mice (Flox-Exos) resulted in significant phenotypic reversion, including down-regulation of genes and proteins involved in adipogenesis. Mechanistically, miR-146a-5p has also been demonstrated to function as a negative regulator of peroxisome proliferator-activated receptor γ (PPARγ) signaling by directly targeting growth and differentiation factor 5 (GDF5) gene to mediate adipogenesis and fatty acid absorption. Taken together, these data provide new insights into the role of miR-146a-5p as a novel myokine involved in the regulation of adipogenesis and obesity via mediating the skeletal muscle-fat signaling axis, which may serve as a target for the development of therapies against metabolic diseases, such as obesity.

## 1. Introduction

Adipose tissue and skeletal muscle are highly heterogeneous endocrine organs that secrete several hormones, with myokines and adipokines participating in local autocrine/paracrine interactions and crosstalk with other tissues [1]. Myokines and adipokines are essential for the maintenance of body muscle and fat levels and modulation of the body composition [2]. Irisin stimulates uncoupling protein 1 (UCP1) expression on white adipose cells in vitro and in vivo, which results in brown-fat-like development, while muscle-specific expression of PPARγ coactivator-1 α (PGC1α) drives browning of subcutaneous white adipose tissue [3]. As reported, the muscle interleukin-6 (IL-6) influences the main neuropeptides for energy homeostasis in a sex-specific manner [4]. The hormone myostatin inhibits myogenesis and promotes adipogenic differentiation of mesenchymal cells [5]. Prolyl hydroxylase 3 (PHD3) losses during endurance exercise challenges improve exercise capacity [6]. In mice with GR mKOs in the skeletal muscle, muscle mass is increased, while fat tissue is smaller [7]. The present study demonstrated that exercise induces myokines to counteract the negative effects of pro-inflammatory adipokines [8]. In recent years, there has been increased interest in investigating the effects of exercise training on adipose tissue [9].

Exosomes are small extracellular vesicles with a diameter of 50–150 nm, which are formed when multivesicular endosomes fuse with the plasma membrane and contained biologically active substances, such as proteins, RNA, DNA, cholesterol, etc. [10,11]. Secreted exosomes are taken up by and deliver their content to the recipient cells, thus representing a novel intercellular communication pathway [12]. Muscle and adipose exosomes can act as a mediator of intercellular communication to exert their physiological regulatory functions. There is increasing evidence that exosomes released by myogenic cells can transport their proteins, mRNAs, and miRNAs to recipient cells and regulate myocyte function in an autocrine or paracrine manner [13]. They can also enter the circulatory system, such as the blood, and may act on distant tissues [14,15]. The incorporation of muscle exosomes into various tissues in vivo, including the pancreas and liver, suggests that skeletal muscle (SKM) could transfer specific signals via the exosomal route to key metabolic tissues [16]. Endocytosis, membrane fusions, and receptor-mediated internalization are the mechanisms by which exosomes are absorbed intracellularly [17]. These variable internalization mechanisms and the signaling molecules presenting in exosomes are the reason why exosomes are widely accepted as important players of intercellular communication in the microenvironment and worthy of investigation [18].

The miRNA gene family adds a new layer of regulation and fine-tuning to gene expression that may affect a wide range of cellular functions, including metabolism, and numerous studies indicate that miRNAs play important roles in diverse aspects of signaling and metabolism, despite their unknown functions [19]. miR-27a released from adipocytes of high-fat diet-fed C57BL/6J mice was associated with a triglyceride accumulation. Exosomal miR-27a derived from adipocytes induces insulin resistance in C2C12 muscle cells through miR-27a-mediated repression of PPARγ and downstream genes involved in obesity [20]. miR-130b’s circulation could act as a metabolic mediator in adipose-muscle crosstalk, as well as a potential contributor to obesity-associated metabolic diseases [21]. MiR-124 secreted by adipose-derived stem cells has been implicated in skin wound healing, possibly by targeting MALAT1 and activating Wnt/catenin signaling pathways [22]. Accumulating evidence indicates that miR-146a-5p is a multifunctional miRNA that can act as a multidirectional target to regulate body metabolism. Mechanistically, miR-146a-5p attenuates TGF-β signaling by directly targeting SMAD family member 4 (SMAD4), thereby inhibiting cell proliferation, and attenuates AKT/mTORC1 signaling by targeting TNF receptor-associated factor 6 (TRAF6) to inhibit the differentiation of intramuscular preadipocytes [23]. Further studies revealed that hepatic miR-146a-5p overexpression significantly improved glucose and insulin tolerance as well as lipid accumulation in the liver by targeting the mediator complex subunit 1 gene (MED1) to promote the oxidative metabolism of fatty acids [24]. In long-living Ame’s dwarf (df/df) mice, miR-146a-5p mimetic treatment increased cellular senescence and inflammation and decreased pro-apoptotic factors in visceral adipose tissue [25]. Furthermore, the miR-146a gene might be a powerful target for preventing age-related bone dysfunctions such as the formation of bone marrow adiposity and osteoporosis [26]. However, whether skeletal muscle-derived exosomes by transferring miRNAs and then affects adipogenesis associated signaling pathways remains elusive.

Recently, we compared the expression profiles of miRNA between exosomes derived from skeletal muscle and adipose tissue [27]. The findings showed that the content of miR-146a-5p in skeletal muscle-derived exosomes was more than 50 times higher than that in fat-derived exosomes, indicating that the miR-146a-5p may play a crucial role in regulating the skeletal muscle-fat axis. In this study, we intend to explore the connection between skeletal muscle and adipose tissue via the mediation of exosomes, especially, miR-146a-5p from skeletal muscle-derived exosomes mediating crosstalk between skeletal muscle and adipose tissue. Through transwell assay, gain-of-function and loss-of-function strategies in cell models, and skeletal muscle-specific miR-146a-5p knockout animal models, in vitro and in vivo studies have gradually revealed the exosomal miR-146a-5p released from skeletal muscle as a new myokine involved in the regulation of adipogenesis via mediating the skeletal muscle-fat signaling axis.

## 2. Results

### 2.1. Transwell Co-Culture of C2C12 Cells Inhibits the Adipogenesis of 3T3-L1 Cells

**Figure 1:**
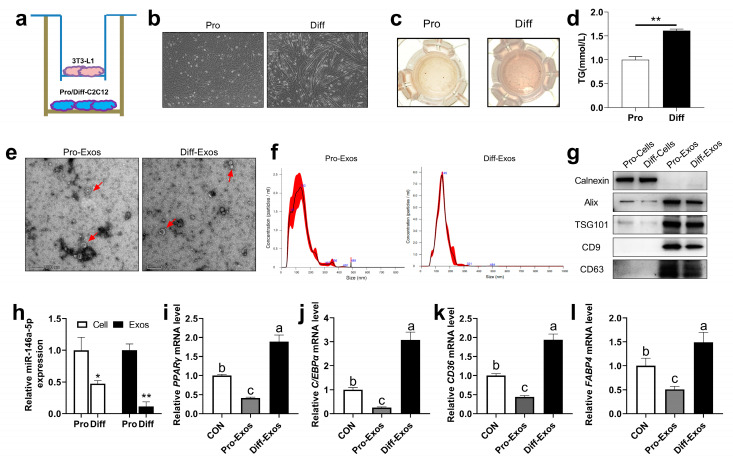
Effects of skeletal muscle-derived exosomes on adipogenesis in 3T3-L1 cells. (**a**) C2C12 cells were co-cultured with 3T3-L1 cells, and the cells were grown in a transwell. (**b**) The morphology of C2C12 cells before and after differentiation was observed under a microscope (scale bar = 100 μm). (**c**) During co-culture, differentiated 3T3-L1 adipogenic precursor cells were induced to mature, and oil red O staining was performed. (**d**) During co-culture, the differentiation of 3T3-L1 adipocyte precursor cells was induced to mature to examine TG content (n = 6). (**e**) Electron microscopy photographs of C2C12 cells-derived exosomes proliferation (Pro-Exos) and differentiation (Diff-Exos) (scale bar = 200 nm). (**f**) Nanoparticle tracking analysis (NTA) was used to determine the size distribution and concentration of exosomes. (**g**) Marker proteins Alix, TSG101, CD9, and CD63 in exosomes extracted from C2C12 cells, and the Western Blot detection bands of endoplasmic reticulum marker protein Calnexin. (**h**) The expression of miR-146a-5p in C2C12 cells proliferation and differentiation (n = 6), and the expression of miR-146a-5p in C2C12 cells-derived exosomes of the proliferation and differentiation (n = 4). (**i**–**l**) After treatment with skeletal muscle-derived exosomes (Pro-Exos, Diff-Exos), adipogenesis-related genes PPARγ and C/EBPα, and fatty acid synthesis-related genes CD36 and FABP4, were detected by real-time quantitative PCR after induced differentiation in 3T3-L1 preadipocytes (n = 6). Values are presented as means ± SEM, * *p <* 0.05, and ** *p <* 0.01, according to the non-paired Student’s *t*-test or one-way ANOVA between individual groups. Different letters mean significant difference (*p* < 0.05), and the same letters mean no significant difference (*p* > 0.05).

To further determine whether muscle cells can regulate adipocyte differentiation and lipid deposition via secreted exosomes, we used the transwell co-culture experiments with C2C12 cells and 3T3-L1 cells to test this possibility (Figure 1a). C2C12 myoblasts during proliferation (Pro) were cultured in vitro and induced to differentiate into mature myofibroblasts (Diff) (Figure 1b). The result showed that the differentiated C2C12 cells promoted the deposition of lipid droplets (Figure 1c), and significantly increased the content of TG in 3T3-L1 cells (Figure 1d). Next, we extracted the exosomes from the proliferation stage (Pro-Exos) and differentiation stage (Diff-Exos), respectively, and determined the morphology of Pro-Exos and Diff-Exos by electron microscopy (Figure 1e); nanoparticle tracking analysis (NTA) showed that the exosomes were mainly concentrated at 130–150 nm (Figure 1f), and the exosome marker proteins such as apoptosis-linked gene 2-interacting protein X (Alix), tumor susceptibility gene 101 (TSG101), CD9, and CD63 were mainly enriched in Pro-Exos and Diff-Exos, while the endoplasmic reticulum marker protein Calexin was mainly enriched in cells (Figure 1g), indicating that the exosomes were successfully extracted. Interestingly, we found that the expression of miR-146a-5p in the C2C12 cells’ proliferation stage was significantly higher than that in the differentiation stage of C2C12 cells, and the same expression level of miR-146a-5p also existed in the secreted exosomes (Pro-Exos and Diff-Exos) (Figure 1h). Then, 3T3-L1 cells were treated with Pro-Exos and Diff-Exos and induced to differentiate. RT-qPCR showed that Pro-Exos inhibited the mRNA levels of adipogenesis-related transcriptional factors PPARγ and C/EBPα (Figure 1i–j), and fatty acid synthesis-related genes CD36 and FABP4 (Figure 1k–l), on the contrary, the results of Diff-Exos treatment are reversed. These results suggest that the co-culture of C2C12 cells can inhibit adipogenesis of 3T3-L1 cells, and the reason may be related to exosomal miR-146a-5p secreted by C2C12 cells.

### 2.2. C2C12 Cells-Derived Exosomes Affect Glucose and Fatty Acid Uptake in 3T3-L1 Cells via Transferring of miR-146a-5p

**Figure 2:**
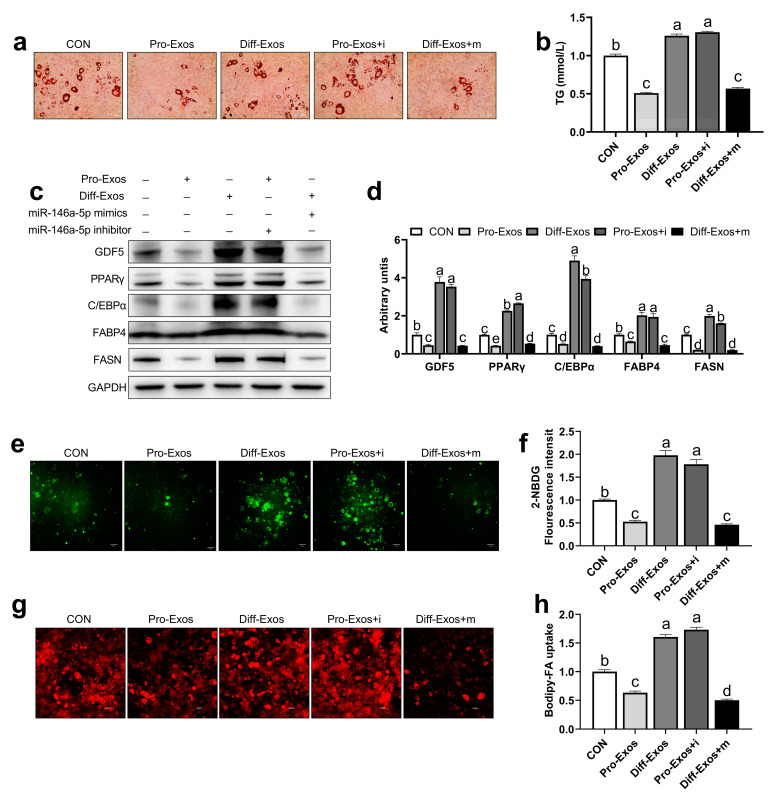
miR-146a-5p reversed the effect of skeletal muscle-derived exosomes on adipogenesis, glucose uptake, and fatty acid absorption in 3T3-L1 cells. (**a**) After treatment of exosomes (Pro-Exos, Diff-Exos) and transfections of miR-146a-5p mimics and miR-146a-5p inhibitor, 3T3-L1 adipogenic precursor cells were induced to mature, and oil red O staining was performed (scale bar = 50 μm). (**b**) After treatment with exosomes (Pro-Exos, Diff-Exos) and transfecting miR-146a-5p mimics (mimics) and miR-146a-5p inhibitor (inhibitor), 3T3-L1 adipocytes were induced to mature, and TG content was assayed (n = 6). (**c**,**d**) After treatment of exosomes (Pro-Exos, Diff-Exos) and transfections of mimics and inhibitor, adipogenesis-related proteins and fatty acid synthesis-related protein bands were detected by Western Blot in mature 3T3-L1 cells (n = 4). (**e**) 2-NBDG fluorescence profile of 3T3-L1 cells after treatment of exosomes (Pro-Exos, Diff-Exos) and transfections of mimics and inhibitor for 24 h (scale bar = 50 μm). (**f**) 2-NBDG fluorescence values of 3T3-L1 cell after treatment of exosomes (Pro-Exos, Diff-Exos) and transfections of mimics and inhibitor for 24h (n = 6). (**g**) Bodipy-FA fluorescence image of 3T3-L1 cells after treatment with exosomes (Pro-Exos, Diff-Exos) and transfections of mimics and inhibitor for 24 h (scale bar = 50 μm). (**h**) Bodipy-FA for 4 h, 3T3-L1 cell fluorescence values after treatment of exosomes (Pro-Exos, Diff-Exos) and transfections of mimics and inhibitor for 24 h (n = 6). Values are presented as means ± SEM, according to the non-paired Student’s *t*-test or one-way ANOVA between individual groups. Different letters mean significant difference (*p* < 0.05), and the same letters mean no significant difference (*p* > 0.05).

To further explore whether skeletal muscle-derived exosomes are involved in regulating adipogenesis and metabolism, especially via miR-146a-5p, we cultured 3T3-L1 adipose precursor cells in vitro to induce their maturation. The results showed that the deposition of lipid droplets and the content of TG in the Pro-Exos treated group was significantly smaller than that of the Diff-Exos group. However, Pro-Exos + i group the miR-146a-5p inhibitor co-treated with Pro-Exos 3T3-L1 cells obtained a similar phenotype to Diff-Exos, indicating that the downregulation of muscle exosomal miR-146a-5p can improve adipogenesis. Similarly, after co-treatment of Diff-Exos with miR-146a-5p mimics (Diff-Exos + m), lipid droplet phenotype and TG content are similar to that of Pro-Exos (Figure 2a,b). Subsequently, the expressions of adipogenesis-related proteins GDF5, PPARγ, C/EBPα, and fatty acid synthesis-related proteins FABP4 and FASN were detected in 3T3-L1 cells of each group. The expression of these proteins was found to be significantly lower in the Pro-Exos and Diff-Exos + m treated groups than in the Diff-Exos and Pro-Exos + i treated groups (Figure 2c,d). To further explore whether skeletal muscle-derived exosomes can affect adipogenesis by affecting glucose and fatty acid uptake in adipocytes, we used fluorescently labeled glucose (2-NBDG) and fatty acids (Bodipy-FA) to observe glycolipid absorption. The amount of glucose absorbed by 3T3-L1 cells in the Pro-Exos treatment group was significantly smaller than that in the Diff-Exos treatment group for the same period. In the Pro-Exos + i group, the glucose uptake of 3T3-L1 cells was significantly greater than that in the Pro-Exos group, and in the Diff-Exos + m group, the absorption of glucose by 3T3-L1 cells was significantly lower than that in the Diff-Exos group (Figure 2e,f). Pro-Exos and Diff-Exos + m treated 3T3-L1 cells had significantly less uptake of free fatty acids than Diff-Exos and Pro-Exos + i treated groups (Figure 2g,h). Experiments showed that C2C12 cells-derived Pro-Exos can inhibit glucose and fatty acid uptake in 3T3-L1 cells, while Diff-Exos can promote glucose and fatty acid uptake in 3T3-L1 cells. Adding the inhibitor and mimics of miR-146a-5p to pro-Exos and diff-Exos, respectively, could reverse the effects of exosomal treatment alone on adipogenesis and glycolipid transport metabolism in 3T3-L1 cells. In summary, the above results highlight the important roles of exosomal miR-146a-5p in mediating the interactions between skeletal muscle cells and the adipocytes’ microenvironment.

### 2.3. miR-146a-5p Significantly Inhibits Adipogenesis, Glucose Uptake and Fatty Acid Absorption in 3T3-L1 Cells

**Figure 3:**
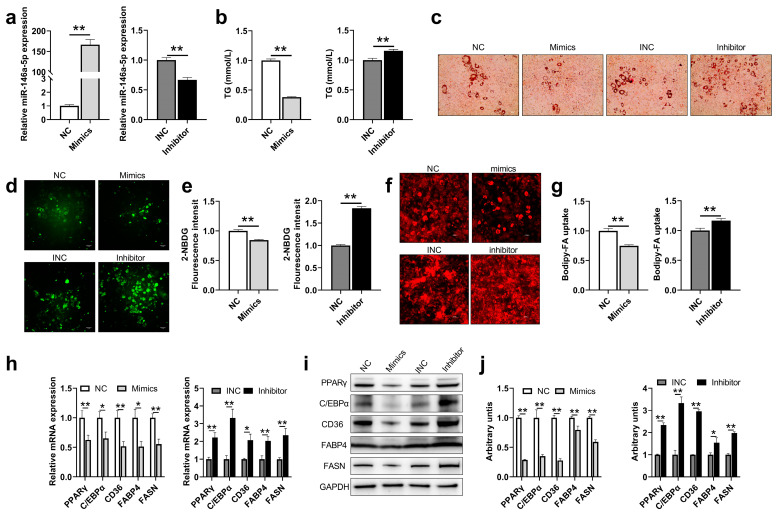
miR-146a-5p was associated with adipogenesis and glucose uptake and fatty acid absorption. (**a**) miR-146a-5p gene expressions in 3T3-L1 adipose mature cells after transfections of miR-146a-5p mimics (Mimics) and miR-146a-5p inhibitor (Inhibitor) (n = 6). (**b**) TG content in 3T3-L1 adipose mature cells after transfections of mimics and inhibitor (n = 6). (**c**) Oil red O staining photographs in 3T3-L1 adipose mature cells after transfections of mimics and inhibitor (scale bar = 50 μm). (**d**) Fluorescence image of 3T3-L1 cells treated with glucose analog 2-NBDG for 1 h after transfections of mimics and inhibitor for 24 h (bar = 50 μm). (**e**) Statistical graph of fluorescence value of 3T3-L1 cells treated with glucose analog 2-NBDG (n = 4). (**f**) Fluorescence image of 3T3-L1 cells treated with free fatty acid analog Bodipy-FA for 4 h after transfections of mimics and inhibitor for 24 h (bar = 50 μm). (**g**) Statistical graph of fluorescence value of 3T3-L1 cells treated with free fatty acid analog Bodipy-FA (n = 4). (**h**) After transfection of mimics and inhibitor adipogenesis-related genes PPARγ, C/EBPα, and fatty acid synthesis-related genes CD36, FABP4, and FASN were detected by real-time quantitative PCR in mature 3T3-L1 (n = 6). (**i**,**j**) After transfection of mimics and inhibitor, adipogenesis-related proteins PPARγ, C/EBPα, and fatty acid synthesis-related proteins CD36, FABP4, and FASN were detected by Western Blot (n = 6). Values are presented as means ± SEM, * *p <* 0.05, and ** *p <* 0.01, according to the non-paired Student’s t-test or one-way ANOVA between individual groups.

To further determine the role of skeletal muscle-derived exosomes in affecting adipogenesis mediated through miR-146a-5p, 3T3-L1 cells were transfected with miR-146a-5p mimics (Mimics) and miR-146a-5p inhibitor (Inhibitor) and induced to mature. The transfection efficiency of miR-146a-5p was quantitatively analyzed first. The expression of miR-146a-5p in 3T3-L1 cells transfected with miR-146a-5p mimics increased 166 times. However, the expression of miR-146a-5p in the miR-146a-5p inhibitor transfected group was also reduced by 33%, and both reached a statistically significant level (Figure 3a). For TG content in each group, it was significantly decreased for miR-146a-5p mimics and significantly increased for miR-146a-5p inhibitor (Figure 3b). At the same time, the results of Oil Red O staining showed that miR-146a-5p mimics could significantly reduce lipid droplet synthesis, while there is a significant increase in miR-146a-5p inhibitor (Figure 3c). To confirm the effect of skeletal muscle-derived exosomes on adipocyte glucose uptake and fatty acid absorption is mediated by miR-146a-5p, we used 2-NBDG and Bodipy-FA to examine the efficiency of glycolipid uptake in 3T3-L1 cells. The 3T3-L1 cells with miR-146a-5p inhibitor treatment significantly increased the uptake of glucose and the absorption of free fatty acids, while miR-146a-5p mimics treatment significantly reduced glucose uptake and free fatty acid uptake (Figure 3d–g). It was found by qPCR that miR-146a-5p mimics could significantly reduce the expression of adipogenesis-related genes PPARγ, C/EBPα, and fatty acid synthesis-related genes CD36, FABP4, and FASN, while miR-146a-5p inhibitor significantly increased the expression levels of these genes (Figure 3h). Western blot results were consistent with the quantitative results that miR-146a-5p mimics significantly decreased the expression of adipogenesis-related proteins PPARγ, C/EBPα, and fatty acid synthesis-related proteins CD36, FABP4, and FASN, while miR-146a-5p inhibitor significantly increased the expression of these proteins (Figure 3i–j). The results showed that miR-146a-5p significantly inhibited the differentiation, glucose uptake, and fatty acid absorption of 3T3-L1 preadipocytes.

### 2.4. miR-146a-5p as a Negative Regulator of PPARγ Signaling by Directly Targeting GDF5 to Inhibit Adipogenesis

**Figure 4:**
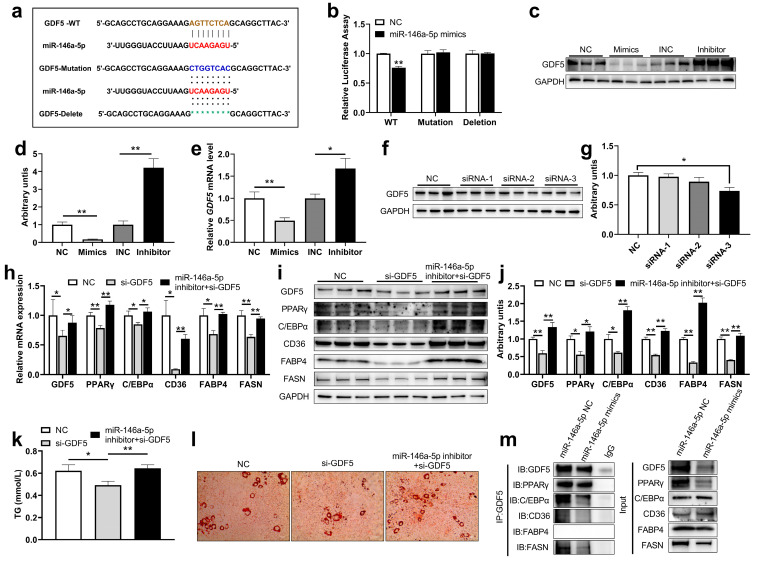
miR-146a-5p regulated PPARγ signaling pathway by targeting GDF5. (**a**) miR-146a-5p has a target interaction with the 3′UTR of GDF5. (**b**) Statistical chart of miR-146a-5p and GDF5 dual luciferase validation fluorescence values (n = 8). (**c**,**d**) After transfections of miR-146a-5p mimics (Mimics) and miR-146a-5p inhibitor (Inhibitor), GDF5 was detected by Western Blot in mature 3T3-L1 cells (n = 6). (**e**) After transfections of mimics and inhibitor, GDF5 gene expression was detected by real-time quantitative PCR (n = 6). (**f**,**g**) 3T3-L1 cells were transfected with GDF5 siRNA for 48 h, and GDF5 was detected by Western Blot (n = 6). (**h**) After transfection (NC, GDF5 siRNA, miR-146a-5p inhibitor + GDF5 siRNA), GDF5, PPARγ C/EBPα, CD36, FABP4 and FASN expressions were detected by real-time quantitative PCR (n = 6). (**i**,**j**) After transfection (NC, GDF5 siRNA, miR-146a-5p inhibitor + GDF5 siRNA), proteins GDF5, PPARγ C/EBPα, CD36, FABP4 and FASN were detected by Western Blot (n = 6). (**k**) After transfection (NC, GDF5 siRNA, miR-146a-5p inhibitor + GDF5 siRNA), TG content was assayed (n = 6). (**l**) After transfection (NC, GDF5 siRNA, miR-146a-5p inhibitor + GDF5 siRNA), oil red O staining was performed (scale bar = 50 μm). (**k**) Oil Red O readings (n = 6). (**m**) Immunoprecipitation assay revealed enrichment of PPARγ C/EBPα, CD36, FABP4, and FASN when introduced with GDF5. Values are presented as means ± SEM, * *p <* 0.05, and ** *p <* 0.01, according to the non-paired Student’s t-test or one-way ANOVA between individual groups.

To determine the targeting mechanism of miR-146a-5p inhibiting adipogenesis, the bioinformatics database miRDB was used to identify putative target genes for miR-146a-5p given the above adipogenesis-related genes and found that miR-146a-5p has a target interaction with the 3′UTR of GDF5 (Figure 4a). Subsequently, the relationship between miR-146a-5p and GDF5 was verified by dual luciferase and miR-146a-5p targeted the 3′UTR of GDF5 and reduced dual-luciferase expression (Figure 4b). In addition, we examined the protein and gene expression changes of GDF5 after miR-146a-5p overexpression and knockdown. As expected, miR-146a-5p overexpression decreased GDF5 protein expression, whereas miR-146a-5p knockdown increased GDF5 protein expression (Figure 4c,d). At the same time, overexpression of miR-146a-5p reduced GDF5 gene expression, and knockdown of miR-146a-5p increased GDF5 gene expression (Figure 4e), which is in line with the trend of miRNA regulation of target genes, and also indicated that miR-146a-5p targeted GDF5. To verify that miR-146a-5p regulates the PPARγ signaling pathway by targeting GDF5, three siRNAs against GDF5 were designed. First, the protein knockdown efficiency of GDF5 siRNA were verified, and GDF5 siRNA-3 significantly reduced GDF5 protein expression (Figure 4f,g). 3T3-L1 cells were transfected with different miR-146a-5p nucleic acid analogs and siRNA (NC, GDF5 siRNA, miR-146a-5p inhibitor + GDF5 siRNA), and the cells were cultured until mature. In 3T3-L1 cells treated with GDF5 siRNA, the expression levels of adipogenesis-related genes GDF5, PPARγ, C/EBPα and fatty acid synthesis-related genes CD36, FABP4 and FASN were significantly decreased, while in those co-treated with GDF5 siRNA and miR-146a-5p inhibitor, the gene expressions of adipogenesis-related genes GDF5, PPARγ, C/EBPα and fatty acid synthesis-related genes CD36, FABP4 and FASN were significantly increased compared with just GDF5 siRNA treatment (Figure 4h). Western blotting results further verified that 3T3-L1 cells transfected GDF5 siRNA significantly reduced the expressions of adipogenesis-related proteins GDF5, PPARγ, C/EBPα, and fatty acid synthesis-related proteins CD36, FABP4, and FASN, while in those co-transfected with miR-146a-5p inhibitor and GDF5 siRNA, the expressions of adipogenesis-related proteins GDF5, PPARγ, C/EBPα and fatty acid synthesis-related proteins CD36, FABP4, and FASN were significantly increased (Figure 4i–j). We found that the content of TG and lipid droplets in the GDF5 siRNA treatment group were significantly lower than NC group, while the co-treatment of GDF5 siRNA and miR-146a-5p inhibitor significantly increased TG and lipid droplet content (Figure 4k–l). Co-immunoprecipitation (co-IP) test further showed that GDF5 has a protein-protein interaction relationship with PPARγ, C/EBPα, CD36, and FASN (Figure 4m). These results suggested that GDF5 participated in adipogenesis by regulating the PPARγ signaling pathway, indicating that miR-146a-5p regulated the PPARγ signaling pathway by targeting GDF5.

### 2.5. Skeletal Muscle-Specific Knockout miR-146a-5p Significantly Increased Body Weight Gain and Decreased Oxidative Metabolism in Mice

**Figure 5:**
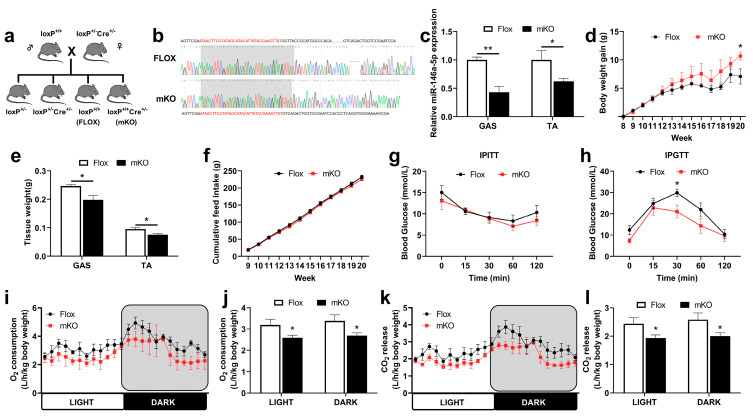
Regulations of growth and metabolism in transgenic mice. (**a**) Mice breeding atlas. (**b**) Sequence alignment of skeletal muscle-specific miR-146a-5p knockout (mKO) and Flox. (**c**) The expression of miR-146a-5p in GAS and TA of mKO mice was detected by quantitative PCR (n = 4). (**d**) Body weight change curve of Flox and mKO mice fed a high-fat diet (n = 4). (**e**) The muscle weight for GAS and TA of Flox and mKO mice fed a high-fat diet (n = 4). (**f**) Accumulate feed intake of Flox and mKO fed HFD (n = 4). (**g**) IPITT blood glucose changes in Flox and mKO mice (n = 4). (**h**) IPGTT blood glucose changes in Flox and mKO mice (n = 4). (**i**,**j**) Oxygen consumption (n = 4). (**k**,**l**) CO_2_ release (n = 4). Values are presented as means ± SEM, * *p <* 0.05, and ** *p <* 0.01, according to the non-paired Student’s *t*-test or one-way ANOVA between individual groups.

To further explore the function of miR-146a-5p, we constructed a skeletal muscle-specific knockout mouse model of miR-146a-5p. Through Sanger sequencing and genotyping results, we confirmed that the mKO mice were successfully constructed (Figure 5a,b). By qPCR, the expression of miR-146a-5p was significantly knocked down in the gastrocnemius (GAS) and tibialis anterior (TA) of mKO mice compared with Flox mice (Figure 5c). Flox and mKO mice were induced with a high-fat diet (HFD) to observe the effect on the growth and metabolism of the mice. During the experiment, it was found that the HFD induction significantly increased the body weight gain of the mKO mice (Figure 5d). We found a significant decrease in muscle mass in both GAS and TA in mKO mice (Figure 5e). However, there was no difference in feed intake (Figure 5f). The skeletal muscle had no significant effect on insulin resistance in miR-146a-5p knockout mice (Figure 5g), but significantly improved glucose tolerance (Figure 5h). In terms of respiratory metabolism, O_2_ inhalation and CO_2_ exhalation in the skeletal muscle of miR-146a-5p knockout mice (mKO) were significantly lower than those in the Flox mice (control group) (Figure 5i–l). To a certain extent, O_2_ inhalation and CO_2_ exhalation reflect the energy metabolism level of the body. Therefore, the experiment showed that the skeletal muscle-specific miR-146a-5p knockout could increase body weight gain and reduce oxidative metabolism in mice.

### 2.6. Skeletal Muscle-Specific Knockout miR-146a-5p Significantly Increased Adipogenesis in Mice by Up-Regulating GDF5 and PPARγ

**Figure 6:**
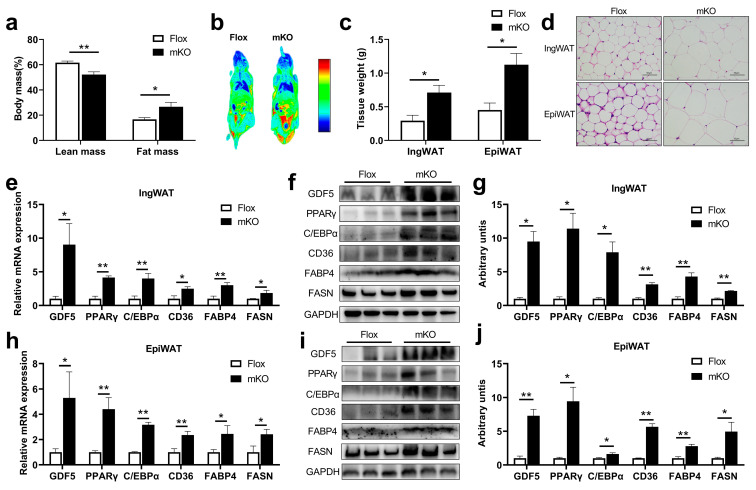
Effects of skeletal muscle-specific miR-146a-5p knockout on adipogenesis in mice. (**a**) Body composition of Flox and mKO mice. (**b**) Body imaging of Flox and mKO mice. (**c**) Tissue weight (IngWAT and EpiWAT) of Flox and mKO mice after feeding with a high-fat diet (HFD) (n = 4). (**d**) HE staining image of IngWAT and EpiWAT in Flox and mKO mice fed with HFD (scale bar = 50 μm). (**e**) Fluorescence quantitative PCR detection of adipogenesis-related genes GDF5, PPARγ, C/EBPα, and fatty acid synthesis-related genes CD36, FABP4, FASN in IngWAT of Flox and mKO mice (n = 4). (**f**,**g**) Western Blot detection of adipogenesis-related proteins GDF5, PPARγ, C/EBPα, and fatty acid synthesis-related proteins CD36, FABP4, and FASN in IngWAT of Flox and mKO mice (n = 4). (**h**) Fluorescence quantitative PCR detection of adipogenesis-related genes GDF5, PPARγ, C/EBPα, and fatty acid synthesis-related genes CD36, FABP4, FASN in EpiWAT of Flox and mKO mice (n = 4). (**i**,**j**) Western Blot detection of adipogenesis-related proteins GDF5, PPARγ, C/EBPα, and fatty acid synthesis-related proteins CD36, FABP4, FASN in EpiWAT of Flox and mKO mice (n = 4). Values are presented as means ± SEM, * *p <* 0.05, and ** *p <* 0.01, according to the non-paired Student’s *t*-test or one-way ANOVA between individual groups.

To further explore how miR-146a-5p knockdown in the skeletal muscle regulates adipogenesis in vivo, the body composition and body imaging of the mice were observed, and the lean mass content of mKO mice was significantly reduced; interestingly, the fat mass content and fat enrichment increased instead, which successfully confirmed the crosstalk in the skeletal muscle and fat axis (Figure 6a,b). For further verification, the tissue weights of inguinal white adipose tissue (IngWAT) and epididymal white adipose tissue (EpiWAT) in mKO mice were found to be significantly higher than those in Flox mice (Figure 6c). At the same time, the HE-stained sections of IngWAT and EpiWAT tissues intuitively revealed that the adipocytes in mKO mice were larger and plumper (Figure 6d). qPCR analysis of adipogenesis-related gene expression showed that the expressions of GDF5, PPARγ, C/EBPα, and fatty acid synthesis-related genes CD36, FABP4, FASN in IngWAT of mKO mice were significantly increased compared with that of Flox mice (Figure 6e). Consistent with the quantitative results, the proteins of these genes were also more highly expressed in the IngWAT tissues of mKO mice (Figure 6f,g). Similarly, in the EpiWAT tissue of mKO mice, the expression of GDF5, PPARγ, C/EBPα, and fatty acid synthesis-related genes CD36, FABP4, FASN was significantly higher than that of Flox mice (Figure 6h), and the protein expressions of these genes were also significantly higher than those of Flox mice (Figure 6i–j). These results suggested that skeletal muscle miR-146a-5p knockout significantly increased adipogenesis in mice.

### 2.7. The Internalization of miR-146a-5p into the mKO Mice by Injecting Flox-Exos Inhibits Adipogenesis

**Figure 7:**
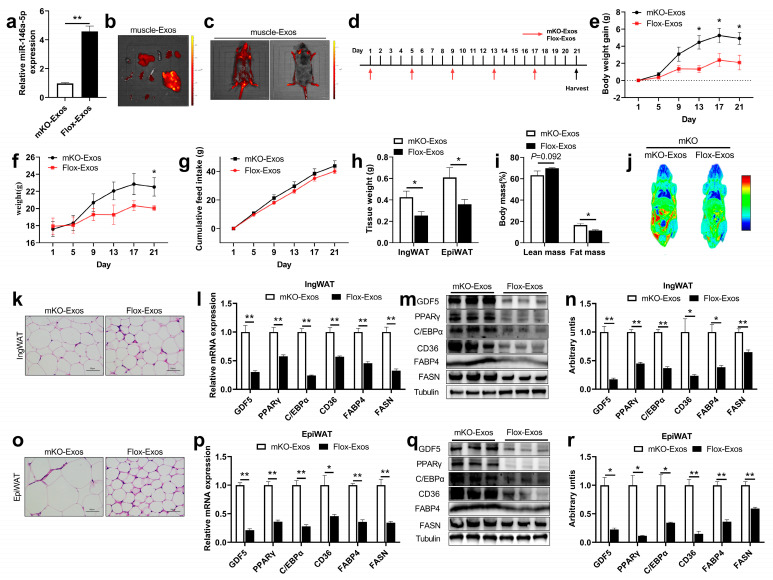
The intravenous injection of Flox-Exos into mKO mice reversed the inhibition of adipogenesis. (**a**) Expressions of miR-146a-5p were determined in skeletal muscle-derived exosomes from mKO and Flox mice (n = 6). (**b**) The fluorescence signal of skeletal muscle-derived exosomes in isolated mKO mice organs 24 h after tail vein injection. The isolated organs from left to right are as follows: heart, liver, spleen, lung, kidney, BAT, IngWAT, EpiWAT, whole intestine, TA, EDL, GAS, SOL. (**c**) In vivo imaging of mKO mice 24h after tail vein injection of PKH67-labeled skeletal muscle-derived exosomes. (**d**) Schematic diagram of tail-vein injections of Flox-Exos and mKO-Exos administered to HFD-fed mKO mice at the age of 6 weeks. (**e**) Body weight gain in mKO mice (n = 3). (**f**) Body weight of mKO mice (n = 3). (**g**) Accumulate feed intake of mKO mice fed HFD (n = 3). (**h**) Tissue weight (IngWAT and EpiWAT) of mKO mice (n = 3). (**i**) Body Composition of mKO mice (n = 3). (**j**) Body imaging of mKO mice. (**k**) IngWAT HE staining image of mKO mice. (**l**) Expression of adipogenesis and fatty acid synthesis-related genes 21 days after exosome injection in IngWAT (n = 3). (**m**,**n**) Expression of adipogenesis and fatty acid synthesis-related proteins in IngWAT (n = 3). (**o**) EpiWAT HE staining image of mKO mice. (**p**) Expression of adipogenesis and fatty acid synthesis-related genes 21 days after exosome injection in EpiWAT (n = 3). (**q**,**r**) Expression of adipogenesis and fatty acid synthesis-related proteins in EpiWAT (n = 3). Values are presented as means ± SEM, * *p* < 0.05, and ** *p* < 0.01, according to the non-paired Student’s *t*-test or one-way ANOVA between individual groups.

To further explore the function of skeletal muscle-derived exosomes, two different skeletal muscle-derived exosomes (Flox-Exos, mKO-Exos) were extracted and identified (Supplementary Figure S1a–c), and the expression level of miR-146a-5p was detected (Figure 7a). The skeletal muscle-derived exosomes were labeled with PKH67 and injected into mice via the tail vein, which was distributed in different organs after 24 h. Interestingly, imaging showed that PKH67-labeled exosomes were mainly distributed in IngWAT, EpiWAT, visceral adipose tissue (VAT), brown adipose tissue (BAT), GAS, TA, liver, lung, kidney (with a small enrichment in extensor digitorum longus (EDL)), soleus (SOL), heart, and spleen (Figure 7b). This indicated that skeletal muscle-derived exosomal miR-146a-5p could be specifically taken up into the fat tissues through humoral circulation (Figure 7c). When mice were continuously injected with skeletal muscle-derived exosomes for 3 weeks (Figure 7d), the body weight gain of Flox-Exos injected mice was significantly reduced at 2 weeks (Figure 7e), and the body weight was also different at 3 weeks (Figure 7f), but there was no difference in feed intake (Figure 7g). After aKO mice were injected with Flox-Exos, IngWAT and EpiWAT tissue weight in mice was significantly reduced (Figure 7h), and the fat mass in body composition decreased significantly, while the lean content showed an increasing trend (Figure 7i), and in vivo imaging also showed fat enrichment was decreased compared to injected with mKO-Exos (Figure 7j). In tissue sections, we found decreased accumulation of lipid droplets in the adipose tissue of mice injected with Flox-Exos (Figure 7k,o). Further studies found that IngWAT adipogenesis and fatty acid synthesis-related mRNA levels were significantly reduced in Flox-Exos-injected mice (Figure 7l), and protein levels were also significantly reduced (Figure 7m,n). Similar results were seen for EpiWAT adipogenesis and fatty acid synthesis-related mRNA and protein levels (Figure 7p–r). These results suggested that miR-146a-5p in skeletal muscle-derived exosomes can be specifically enriched in the adipose tissue, further affecting adipogenesis. Taken together, SKM-Exos-mediated intercellular miR-146a-5p has great potential for the prevention and treatment of obesity.

## 3. Discussion

By binding the 3′UTR of mRNA, miRNAs regulate metabolic homeostasis by repressing or degrading the translation of target mRNA [28]. miR-146a is lowered in obese and type 2 diabetic patients, and mice fed a high-fat diet (HFD) show exaggerated weight gain, increased adiposity, hepatosteatosis, and dysregulated blood glucose levels compared to wild-type controls [29]. miR-146a may be involved in the regulation of inflammation in orbital fibroblasts, contributing to GO pathogenesis [30]. Exosomes derived from miR-146a-modified ADSCs reduced acute myocardial infarction (AMI)-induced myocardial damage by downregulating early growth response factor 1 (EGR1) [31]. Both in vitro and in vivo, miR-146a negatively regulates osteogenesis and bone regeneration in ADSCs [32]. In WAT, miR-146a may contribute to the regulation of inflammatory processes and prevent an overreaction to inflammation [33]. However, some studies suggest that miR-146a deficiency increases inflammation in the liver tissue without affecting lipid deposition in the liver [34]. Our findings indicate that low-abundance miR-146a-5p skeletal muscle-derived exosomes could be circulated to adipose tissue and increase adipogenesis. This indicates that miR-146a-5p plays different roles in different organs.

The interaction between skeletal muscle and fat is dynamic, in which excessive accumulation of fat can cause skeletal muscle atrophy that in turn increases fat differentiation [35]. Adipose tissue is an important fuel reservoir for animal bodies, providing energy for energy-consuming tissues such as the skeletal muscle and ensuring the normal energy operation of the body. The exosome is a natural vehicle for intercellular communication that can penetrate tissues, and diffuse into the blood [36]. These exosomes carry proteins, mRNA, and miRNA for mediating intercellular communication and regulating the function of the recipient cells [37,38]. Previous studies have shown that miR-146a-5p mimics inhibit the proliferation and differentiation of porcine intramuscular adipocyte precursor cells, whereas miR-146a-5p inhibitors promote cell proliferation and adipogenic differentiation of adipogenic precursor cells [23]. After miR-146a-/- systemic knockout, mice were fed a high-fat diet, and their body weight gain was significantly higher; additionally, the sliced cells in the adipose tissue of the mice were significantly larger than those of the control group [39], which is consistent with the phenotype of muscle-specific miR-146a knockout mice in this study. Our study demonstrated that after knocking out miR-146a-5p in mouse skeletal muscle tissue, the adipose tissue showed a promotion effect the same as a miR-146a-5p inhibitor on adipogenic formation, revealing that the skeletal muscle tissue has a potential regulatory effect on adipose development, and the skeletal muscle-derived exosome is the bridge between the two tissues. Thus, the miR-146a-5p is critical for regulating the balance between normal skeletal muscle development and adipogenesis.

In this study, the target gene of miR-146a-5p, GDF5, is a member of the TGF-β superfamily. GDF5 is mainly expressed in developing joints and lateral edges of joints and is a key regulator of cartilage and bone formation [40,41,42]. In addition, GDF5 also plays a key role in embryogenesis, limb development, and connective tissue repair [43]. Overexpression of GDF5 promotes brown adipocyte development in a transgenic mouse model [44]. PPARγ is the main regulatory gene of adipocyte proliferation and differentiation, which promotes adipocyte differentiation and increases the expression of lipid metabolism-related genes. As an important marker gene of adipose differentiation, PPARγ is of great significance to study the regulation of miRNAs. Up to now, a large number of studies have reported that miRNAs can directly or indirectly target the PPARγ signaling pathway to regulate lipid metabolism [45,46]. Subsequent studies found that there is a positive correlation between the gene and protein expressions of GDF5 and PPARγ during the differentiation of 3T3-L1 cells [47]. As research continues, the types of miRNAs found that regulate the expression of PPARγ have increased, and their regulatory mechanisms are gradually explored, providing more selectivity for future applications such as obesity treatment. The present study thus shows that miR-146a-5p internalization plays a critical role in SKM-Exos-mediated of adipogenesis, although other signaling pathways being regulated by GDF5/PPARγ-related cannot be completely excluded.

## 4. Materials and Methods

### 4.1. Animals

The mice were housed under a 12 h light/12 h dark cycle at a constant temperature (23 ± 2 °C) with free access to food and water. The animals were fed ad libitum with standard mouse chow (18% protein, 4.5% fat, and 58% carbohydrate, purchased from Guangdong Medical Science Experiment Center, Guangzhou, Guangdong, China) for the first 8 weeks and high-fat chow (60 kcal% Fat from Research Diets, Cat No. D12492) from 8 to 20 weeks. All animal experiments used female mice aged 20 weeks at the time when they were sacrificed. The miR-146a-5p flox/flox (miR-146a^flox+/+^) and Myf5-Cre mice using CRISPR/Cas9/Cre method were generated (Cyagen, Suzhou, China) and maintained on a C57BL/6 background. They were used to study the metabolic effects of long-term HFD supplementation. To generate skeletal muscle-specific miR-146a-5p knockout (mKO) mice, miR-146a^flox+/+^ mice were first crossed with Myf5-Cre mice to obtain F1(miR-146a^flox+/−,Cre+/−^). Then the F1 mice mated with miR-146a^flox+/+^ mice to produce the mKO mice (miR-146a^flox+/+,Cre+/−^). The primer sequence of mouse genotype identification is shown in Table S1. The care of all animals and procedures at South China Agricultural University complies with “The Instructive Notions with Respect to Caring for Laboratory Animals” issued by the Ministry of Science and Technology of the People’s Republic of China and approved by the Animal Subjects Committee of South China Agricultural University.

### 4.2. NMR Analysis of the Whole-Body Composition

Body composition of mice was determined using quantitative magnetic resonance (QMR, Niumag Corporation, Shanghai, China).

### 4.3. IPITT and IPGTT

Before the intraperitoneal glucose tolerance test (IPGTT), mice fasted for 12 h. By using a blood glucose meter, blood glucose levels were measured at 0, 15, 30, 60, and 120 min after glucose (1 g∙kg^−1^) was injected intraperitoneally. In the intraperitoneal insulin tolerance test (IPITT), mice fasted for 6 h prior to the experiment. Insulin (0.7 U∙kg^−1^) was injected, and blood glucose levels were measured at 0, 15, 30, 60, and 120 min after injection.

### 4.4. In Vivo Oxygen Consumption Assay

Utilizing the promotion metabolism measurement system (Sable Systems International, North Las Vegas, NV, USA), we analyzed O_2_ consumption (VO_2_) and CO_2_ production (VCO_2_) in HFD.

### 4.5. Imaging Experiments

The tissue distribution of PKH67 (Sigma-Aldrich) labeled exosomes were visualized using fluorescence parameters as detected by the IVIS Lumina LT SeriesIII^®®^ imaging system after injection of PKH67-labeled exosomes into the tail vein. We isolated all tissue samples within a 1-h post-mortem, rinsed them in cold PBS to remove blood, and observed them. Exosomes labeled with PKH67 (Sigma-Aldrich, St. Louis, MI, USA) were measured at 490 nm and 520 nm.

### 4.6. HE Staining

Briefly, an aliquot of IngWAT (inguinal white adipose tissue) and EpiWAT (epididymal white adipose tissue) was fixed with 10% formalin and embedded with paraffin. Then, fixed IngWAT and EpiWAT were sectioned and stained with hematoxylin-eosin (HE).

### 4.7. Cell Lines, Culture Conditions, Transfection

The 3T3-L1 cells were grown in high-glucose Dulbecco’s Modified Eagle Medium (DMEM, Gibco) with 10% fetal bovine serum (FBS, Gibco) and 1% penicillin-streptomycin (P/S, Gibco) in a 5% CO_2_ atmosphere at 37 °C. The differentiation was induced by incubating confluent cells (in 12-well plates) for 2 days in differentiation media, which was comprised of DMEM supplemented with 10% FBS, 0.5 mM 3-isobutyl-1-methylxanthine (IBMX), 1 μM dexamethasone, and 10 μg/mL of insulin. Then the cells were cultured with 10 μg/mL insulin in 10% FBS medium by changing the medium every 2 days until a mature lipid droplet appeared. For miR-146a-5p mimics or miR-146a-5p inhibitor (40 nM) (GenePharma, Shanghai, China) or si-GDF5 (50 nM) (Tsingke, Beijing, China) or exosome (10 μg/mL) transfection, 3T3-L1 cells were plated in 12-well dishes at a density of 1.0 × 10^5^ per well and lipofectamine 2000 (Thermo Fisher, Waltham, MA, USA) transfection started at the cell density reached 60 to 70%. The sequence of siRNA transfected by 3T3L-1 cells is shown in Table S2.

### 4.8. Cell Co-Culture

Transwell chambers (BIOFIL, TCS016012) were used to construct the co-culture systems. At the beginning of the test, the upper layer of the cell chamber was inoculated with 3T3-L1 cells (2.0 × 10^4^ cells per well), and the lower layer with C2C12 cells (8.0 × 10^4^ cells per well). They were cultured separately, co-cultured, and contacted for 48 h to detect indicators.

### 4.9. Collection of C2C12 Cell Culture Medium Supernatant

The C2C12 cells were seeded in 75 cm^2^ cell culture flasks (1.0 × 10^6^ cells/flasks) (Corning, Corning, NY, USA) exosome-free 10% FBS DMEM and grown for 48 h. Then, the cellular supernatant was collected. The C2C12 cells were plated in 12-well plates (Corning, 3513), seeded with 8.0 × 10^4^ cells per well in DMEM supplemented with 10% FBS and 1% P/S. By adding 2% horse serum (HS, Gibco) after reaching 80% confluency, C2C12 cells became myotubes for 4 days. The supernatant was collected by contacting the cells with 2% exosome-free HS DMEM for 48 h.

### 4.10. Collection of Skeletal Muscle Tissue Culture Medium Supernatant

In order to obtain the mice skeletal muscle tissue-derived exosomes, the mice’s were first identified by genotype. After identification, Flox mice and mKO mice were sacrificed by cervical dislocation and immersed in a beaker of 75% alcohol and isolated in a sterile environment. The skeletal muscle tissue of mice was removed, washed with PBS (containing 1% P/S), and placed in a medium containing exosome-free 10% FBS DMEM. Then, the skeletal muscle tissue was carefully cut into 1 mm^3^ pieces with fine scissors for 10 min and washed with PBS 3 times. Then, they were placed in a petri dish and kept in an incubator of a 5% CO_2_ atmosphere at 37 °C for 24 h to collect the medium supernatant. The exosomes were named Flox-Exos and mKO-Exos, respectively.

### 4.11. Exosome Isolation

Isolation of exosomes in culture supernatant by ultracentrifugation. The specific steps were as follows. After centrifuging the culture supernatant at 2000× *g* for 10 min and 12,000× *g* for 30 min, large debris and dead cells were removed. An ultracentrifuge of 100,000× *g* for 70 min was performed on the supernatant. Finally, the cells were rinsed in 38 mL PBS and ultracentrifuged for 70 min at 100,000× *g*. We resuspended the pellets in 100 μL of PBS and stored them at −80 °C.

### 4.12. Transmission Electron Microscopy Analysis

Exosomes of 10 μL were placed on copper grids coated with formvar, incubated for 5 min, and excess liquid was discarded. Uranyl acetate was added to the grid for negative stain for 1 min, and excess liquid was discarded. At 100 kV, samples were examined using transmission electron microscopy.

### 4.13. Dual-Luciferase Reporter Experiments

We seeded HEK293T cells in 96-well plates (Corning) at 2.5 × 10^4^ cells per well and grew them overnight to 70–80% confluence. The dual-luciferase reporter plasmids were co-transfected with miRNA into HEK293T cells and the dosage of miR-146a-5p NC/mimic and wild-type/mutation/deletion dual-luciferase gene reporter vector per well was 3 pmol and 100 ng, respectively. The Dual-Luciferase Reporter Assay System (Promega) was used to detect luciferase activity after 48 h.

### 4.14. Nanoparticle Tracking Analysis

The exosomes were diluted to appropriate concentrations with PBS. The size of exosomes derived from cells or skeletal muscle tissue was measured by nanoparticle tracking analysis (NTA). Refer to the manual for the specific use of the instrument, including sample loading, photo-taking, and result statistics in brief.

### 4.15. Co-IP Experiment

The specific steps are shown in the instructions. In short, Pierce™ Protein A/G Magnetic Beads (88803, Thermo Scientific, Waltham, MA, USA) were used to bind GDF5 antibody and added to the lysed samples. The magnetic beads were pulled down with a magnet, and the resulting precipitate was detected using a western blot to confirm whether the target protein exists, and the sample lysate was directly used as the Input group control to detect the target protein.

### 4.16. Oil Red O Staining

After being treated and induced to mature, 3T3-L1 cells (24-well plates, Corning) were washed 3 times with PBS buffer, fixed in 4% formaldehyde for 30 min at room temperature, washed 3 times with PBS for 5 min each, and then stained with oil red O (Sigma-Aldrich, Shanghai, China) for 1 h. To create a working solution, oil red O was first diluted with water (3:2) and filtered through filter paper. After staining the cells, the plates were washed 3 times in PBS for 5 min each and then photographed under a microscope (TE2000-E; Nikon, Japan).

### 4.17. Triglyceride Accumulation

Triglyceride (TG) content was determined using a colorimetric/fluorometric assay kit (Biovision, Milpitas, CA, USA). 3T3-L1 cells were seeded into a 96-well plate and differentiated with CTE (500–1000 µg/mL) until they became mature adipocytes. A lipid droplet was then extracted by extraction buffer and converted by lipase to glycerol and fatty acid. A wavelength of 570 nm was used to measure the released glycerol.

### 4.18. Fatty Acid and Glucose Uptake Assay

Fatty acid and glucose uptake assays were carried out using Bodipy-FA (Invitrogen Cat *No.* D3835) and 2-NBDG (Sigma Cat No. 186689-07-6), which are fluorescent tracers.

### 4.19. Quantitative Real-Time PCR

We extracted the total RNA using TRIzol (Thermo Fisher). 1 µg of RNA was converted into complementary deoxyribonucleic acid (cDNA) using Color Reverse Transcription Kit (EZBioscience, Roseville, MN, USA, Cat No. A0010CGQ). We performed quantitative real-time PCR (qPCR) using a QuantStudio Real-Time PCR System (Bio-Rad C1000 Touch) using 2 × RealStar Fast SYBR qPCR Mix (GenStar, Cat No. A301). The mRNA and miRNA internal references were GAPDH and U6. Quantitative real-time PCR primer sequence is shown in Table S3, and reverse transcription primer sequences are shown in Table S4.

### 4.20. Protein Extraction and Western Blot Analysis

Radioimmunoprecipitation assay (RIPA) buffer containing protease and phosphatase inhibitors (BestBio Cat No. BB-3101) was used to extract proteins. The protein concentration was assessed using the Rapid Gold BCA Protein Assay Kit (Thermo Fisher). Western blotting analysis was performed by loading 15 µg of lysate onto sodium dodecyl sulfate-polyacrylamide gel electrophoresis (SDS-PAGE) gels, transferring the gels to polyvinylidene difluoride (PVDF) membranes (Millipore), and incubated with rabbit anti-GDF5 (1:1000, #A13167; ABclonal), rabbit anti-PPARγ (1:1000, #2443; CST), rabbit anti-FASN (1:1000, #D262701; Sangon Biotech), rabbit anti-C/EBPα (1:1000, #2295; CST), rabbit anti-FABP4 (1:1000, #2120; CST), rabbit anti-CD36 (1:1000, #ab1336-25; Abcam), rabbit anti-GAPDH (1:5000, #BS65529; Bioworld), rabbit anti-CD9 (1:1000, #AP68-965-100; Abcepta), rabbit anti-CD63 (1:2000, #D160973; Sangon Biotech), rabbit anti-TSG101 (1:2000, #381538; ZEN BIO), rabbit anti-Alix (1:1000, #D262028; Sangon Biotech) or rabbit anti-Calnexin (1:1000, #D262986; Sangon Biotech). Afterward, goat anti-rabbit secondary antibody (1:50000, # BS13278, Bioworld) conjugated with HRP was used. GAPDH levels served as the loading control. The amount of protein was measured using ImageJ software.

### 4.21. Statistical Analysis

SPSS 25 and Graphpad prism 9.0 were used for one-way ANOVA and stand-alone sample t-test analysis and plotting. The results were presented as mean ± standard error of the mean (SEM). The significance of the difference was judged by a level of * *p* < 0.05 or ** *p* < 0.01. The letters a, b, and c represent the level of statistical significance of the difference between the groups. Different letters mean a significant difference, and the same letters mean the difference is not significant.

## 5. Conclusions

In conclusion, our results suggest that high levels of miR-146a-5p in mice are inversely correlated with adipogenesis. Skeletal muscle secreted large quantities of exosomes containing abundant proteins, mRNA, and miRNAs. Additionally, there were notably high levels of miR-146a-5p. Under the uptake of adipocytes to skeletal muscle-derived exosomes, the skeletal muscle exosomal miR-146a-5p inhibits the synthesis of lipid droplets and adipocyte differentiation by down-regulating the expression of GDF5 in adipocytes and repressing the PPARγ signaling pathway. In addition, miR-146a-5p blocked fatty acid uptake by decreasing CD36 expression. miR-146a-5p-specific knockout in skeletal muscle can improve the body weight, fat ratio, and glucose tolerance, and reduce the body’s oxidative respiratory metabolism in mice. Our study provides new insights into the role of miR-146a-5p as a novel myokine in the cross-talk between skeletal muscle and fat tissue and contributes to the prevention and improvement of obesity by maintaining an appropriate ratio of skeletal muscle to fat.

## Data Availability

Not applicable.

## Supplementary Materials

The following supporting information can be downloaded at: https://www.mdpi.com/article/10.3390/ijms24054561/s1.

## Abbreviations

CD36: Cluster of differentiation 36 receptor; C/EBPα: CCAAT/enhancer binding protein alpha; EpiWAT: Epididymal white adipose tissue; Exos: Exosomes; FABP4: Fatty acid binding proteins4; Flox-Exos: Skeletal muscle-derived exosomes from the Flox mice; GDF5: Growth and differentiation factor 5; HE: Hematoxylin-eosin; IngWAT: Inguinal white adipose tissue; IPGTT: Intraperitoneal glucose tolerance test; IPITT: Intraperitoneal insulin tolerance test; miRNAs: microRNAs; mKO: skeletal muscle-specific knockout miR-146a-5p; NTA: Nanoparticle tracking analysis; PPARγ: Peroxisome proliferator-activated receptor γ; SKM: Skeletal muscle; SKM-Exos: Skeletal muscle-derived exosomes; TG: triglyceride.
