# Supplementary material for: Skeletal Muscle-Derived Exosomal miR-146a-5p Inhibits Adipogenesis by Mediating Muscle-Fat Axis and Targeting GDF5-PPARγ Signaling
Source: Int J Mol Sci. 2023 Feb 25;24(5):4561. doi: 10.3390/ijms24054561 (PMC10003660; doi:10.3390/ijms24054561)
Supplement: Supplementary file 1 [file ijms-24-04561-s001.zip › ijms-2207905-supplementary.pdf]

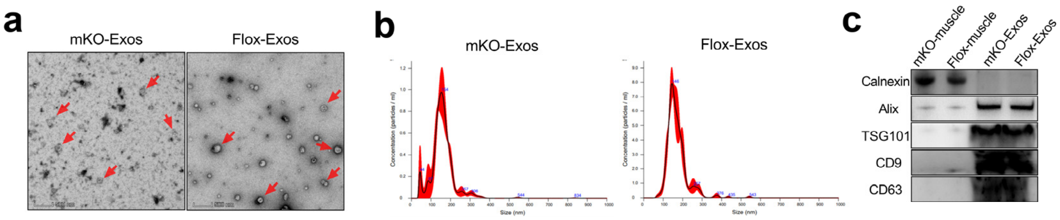


**Supplementary Figure S1 Exosome identification of mouse skeletal muscle tissue.** (a) Electron microscopy results of skeletal muscle-derived exosomes in mKO and Flox mice (scale bar = 500 nm). (b) Nanoparticle tracking analysis was used to determine the size distribution of exosomes. (c) The marker protein Calnexin in skeletal muscle cells of mKO and Flox mice and the marker proteins Alix, TSG101, CD9 and CD63 in exosomes were detected by Western Blot.

**Table S1. Primer sequences for genotypic identification**

| Gene | Forward (5'-3') | Reverse (5'-3') |
|---|---|---|
| Loxp-5 | CTGCTCTTGCTGACGTGAAGAA | TTCCTAGAGTGACCCAGTTCTACATG |
| Loxp-6 | GATGATCCCTCACTAACACTCTTTCTA | GAGTCACAGCAGCAAGAACCACTC |
| Myf5-Cre | ACGAAGTTATTAGGTCCCTCGAC | CGGCTCTTAAAGCAATGGTC |

**Table S2. siRNA sequences for 3T3L-1 cells transfection**

| Gene | Forward (5'-3') | Reverse (5'-3') |
|---|---|---|
| GDF5 siRNA-1 | CACCAGCUUUAUUGACAAATT | UUUGUCAAUAAAGCUGGUGTT |
| GDF5 siRNA-2 | GACCUGUUCUUUAAUGAGATT | UCUCAUUAAAGAACAGGUCTT |
| GDF5 siRNA-3 | GCAAGGCCUUGCAUGUCAATT | UUGACAUGCAAGGCCUUGCTT |
| mmu-miR-146a-5p mimics | UGAGAACUGAAUUCCAUGGGUU | CCCAUGGAAUUCAGUUCUCAUU |
| mmu-miR-146a-5p inhibitor | AACCCAUGGAAUUCAGUUCUCA | |

**Table S3. Primer sequences for quantitative real-time PCR**

| Gene | Forward (5'-3') | Reverse (5'-3') |
|---|---|---|
| mmu-GAPDH | AGGTCGGTGTGAACGGATTTG | TGTAGACCATGTAGTTGAGGTCA |
| mmu-U6 | CTCGCTTCGGCAGCACA | AACGCTTCACGAATTTGCGT |
| mmu-miR-146a-5p | GGGTGAGAACTGAATTCCA | CAGTGCGTGTCGTGGAGT |
| mmu-GDF5 | AGCAGCGTGAAGTTGGAGG | CTTCCGTAAGATCCGCAGTTC |
| mmu-PPARγ | CGCTGATGCACTGCCTATGA | AGAGGTCCACAGAGCTGATTCC |
| mmu-FASN | GGAGGTGGTGATAGCCGGTAT | TGGGTAATCCATAGAGCCCAG |
| mmu-CD36 | AGATGACGTGGCAAAGAACAG | CCTTGGCTAGATAACGAACTCTG |
| mmu-C/EBPα | GCGGGAACGCAACAACATC | GTCACTGGTCAACTCCAGCAC |
| mmu-FABP4 | ATCAGCGTAAATGGGGATTTGG | GTCTGCGGTGATTTCATCGAA |

**Table S4. Primer sequences for reverse transcription**

| Gene | Sequences (5'-3') |
|---|---|
| mmu-miR-146a-5p RT | GTCGTATCCAGTGCGTGTCGTGGAGTCGGCAATTGCACTGGATACGACAACCCA |
